# Polyoxometalates on Functional Substrates: Concepts, Synergies, and Future Perspectives

**DOI:** 10.1002/advs.201903511

**Published:** 2020-03-06

**Authors:** Alexey S. Cherevan, Sreejith P. Nandan, Isolda Roger, Rongji Liu, Carsten Streb, Dominik Eder

**Affiliations:** ^1^ Institute of Materials Chemistry Vienna University of Technology Getreidemarkt 9/BC/02 Vienna 1060 Austria; ^2^ Institute of Inorganic Chemistry I Ulm University Albert‐Einstein‐Allee 11 Ulm 89081 Germany; ^3^ CAS Key Laboratory of Green Process and Engineering Institute of Process Engineering Chinese Academy of Sciences Beijing 100190 China; ^4^ Helmholtz‐Institute Ulm Helmholtzstr. 11 Ulm 89081 Germany

**Keywords:** charge transfer, composites, heterogenization, molecular metal oxides, substrates

## Abstract

Polyoxometalates (POMs) are molecular metal oxide clusters that feature a broad range of structures and functionalities, making them one of the most versatile classes of inorganic molecular materials. They have attracted widespread attention in homogeneous catalysis. Due to the challenges associated with their aggregation, precipitation, and degradation under operational conditions and to extend their scope of applications, various strategies of depositing POMs on heterogeneous substrates have been developed. Recent ground‐breaking developments in the materials chemistry of supported POM composites are summarized and links between molecular‐level understanding of POM‐support interactions and macroscopic effects including new or optimized reactivities, improved stability, and novel function are established. Current limitations and future challenges in studying these complex composite materials are highlighted, and cutting‐edge experimental and theoretical methods that will lead to an improved understanding of synergisms between POM and support material from the molecular through to the nano‐ and micrometer level are discussed. Future development in this fast‐moving field is explored and emerging fields of research in POM heterogenization are identified.

## Graphical abstract

Recent ground‐breaking developments in the materials chemistry of supported polyoxometalates (POMs) are summarized. Links between a molecular‐level understanding of POM‐support interactions and macroscopic effects including new or optimized reactivity, improved stability, and added functions are established.

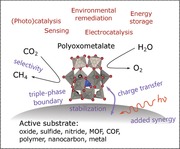

## 1 Introduction

Over the last decades, polyoxometalates (POMs) have captured the imagination of chemists and material scientists, as they combine the chemical properties of solid‐state oxides with the chemical versatility of molecular components. POMs are molecular metal oxide anions based on early, high‐valent transition metals (M often Mo, W, V). The POM family forms the link between small oxometalate species and bulk solid‐state metal oxides. Their wide structural and chemical tunability, together with their unique properties (acidity, redox‐activity, photoactivity, etc.), makes them ideal models to explore metal oxide reactivity at the molecular level.

In particular so‐called hetero‐POMs, [X_a_ *M′*_b_M_c_O_d_]*^n^* ^−^ are amenable to chemical tuning, as their structure and properties can be modified by variation of the central heteroatom template X (often an oxo‐anion, e.g., XO_4_ *^n^* ^−^; X = B, Si, P, etc.) and by incorporation of heterometals *M′* from throughout the periodic table into the cluster framework.^[^ ^1^, ^2^, ^3^ ^]^ In addition, important material properties such as solubility can be tuned by variation of the corresponding counter‐cation.^[^ ^4^ ^]^

Early application studies of POMs investigated their Bronsted‐acidity as well as their oxidative and photooxidative reactivity, typically under homogeneous conditions. These conditions are ideally suited to enable mechanistic analyses and in addition allow exposure of all POM molecules to other reagents, thereby ensuring maximum reactivity. For these processes, POMs feature several appealing properties including cation‐controlled solubility in aqueous and organic solvents, high Bronsted‐acidity and multiple protonation sites (e.g., [H_3_PW_12_O_40_]); high redox‐activity and oxygen transfer capability together with high stability under harsh conditions^[^ ^5^ ^]^ and high photoactivity in the UV and near‐visible region.^[^ ^6^ ^]^ A number of reviews have summarized the use of POMs for homogeneous catalysis including organic substrate oxidation, C—H activation, photooxidations, and water oxidation reactions.^[^ ^6^, ^7^, ^8^ ^]^

While homogeneous catalysis opened the door for advanced technological processes based on POMs, challenges associated with this reactivity including aggregation, precipitation, loss of activity and difficult separation led to early studies that explored the use of POMs as heterogeneous catalysts.^[^ ^9^ ^]^ Initially, bulk solid POMs were studied as heterogeneous acid catalysts and oxidation catalysts. However, this approach is often affected by low specific surface areas, leading to reduced reactivity.^[^ ^10^ ^]^ Later studies, therefore, pioneered the immobilization of POMs on high surface‐area substrates including porous silica and other metal oxides,^[^ ^10^ ^]^ as well as more advanced materials such as carbon nanostructures^[^ ^11^ ^]^ and metal–organic frameworks (MOFs).^[^ ^9^ ^]^ This strategy can enable maximized exposure of the individual POM molecules to the reaction environment, and in addition, allows for facile separation by filtration or centrifugation.^[^ ^12^ ^]^

While early studies on POM immobilization used heterogeneous supports for mechanical stabilization and maximized surface‐area, more recent studies have moved toward added properties introduced by the support. These include light‐absorption and charge transport by semiconductors (TiO_2_, CdSe, etc.), electrical conductivity (by carbons, metals, conductive polymers) as well as specific POM binding sites, as introduced in MOFs and organo‐functionalized supports. Many contemporary studies in this field share a common interest in exploring the synergism between POM and substrate to tune and optimize reactivity and stability of the composite. The concept has been theoretically and experimentally described several decades ago as the so‐called “microenvironment effect” (electronic charge enhancement) where entrapment of POM species in a 3D matrix has been shown to induce huge improvement of their electrocatalytic activity.^[^ ^13^, ^14^, ^15^ ^]^ However, to‐date, the understanding of the fundamental processes which govern synergism of POM‐substrate interactions is still in its infancy, as molecular‐level understanding of these complex processes by experiment or theory is far from trivial. This is due to the vast number of materials combinations, which are currently studied, and is further complicated by the often unknown chemical structures of the POM‐substrate interface which make theoretical studies difficult. Also, in situ/operando studies of these materials are still challenging, and instrumentational approaches to address these issues are still being developed and not widely available. Most current experimental studies, therefore, focus on macroscopic reactivity, while providing less insight into the underlying causes of the processes observed.

This Progress Report aims at raising awareness of the vast benefits of exploring this under‐researched area to enable full use of POM‐substrate interactions including charge and energy transfer, band‐gap, and frontier orbital alignment as well as interface design for stability and reactivity. These fundamental concepts can affect both the thermodynamics as well as the kinetics of the chemical processes studied so that new reactivity can be tailored by understanding of the interactions between POM and substrate. Given the fast‐paced progress in the synthesis, application, and characterization of this materials class, this Progress Report will serve as a focal point for the community to draw attention to the most pressing challenges, identify areas of widespread research interest, and propose emerging areas which could shape the future of the field.

Over the last years, several reviews have focused on specialized aspects of POM heterogenization; however, none of these reviews addressed substrate effects, nor did they include a specific discussion of POM‐substrate interactions or analyzed differences in performance between homogeneous and heterogenized POMs. In 2014, Zhou et al. provided a comprehensive overview of different POM heterogenization routes with a focus on recent advances in liquid‐phase catalytic applications.^[^ ^12^ ^]^ In 2015, Ji et al. summarized developments in POM‐nanocarbon composites with a focus on (electro)catalysis, energy storage and sensing.^[^ ^11^ ^]^ In 2015, Herrmann et al. reviewed POM‐conductive polymer composites and their applications.^[^ ^3^ ^]^ In 2016, Ye et al. provided an overview of the design, synthesis, and catalytic properties of POM‐based solids including widely investigated POM‐based MOFs as well as POM crystals and organic–inorganic POM hybrids.^[^ ^9^ ^]^ In 2019, Chen et al. reviewed the progress of POM‐containing dye‐sensitized solar cells.^[^ ^16^ ^]^

In contrast to these existing reviews, the aim of this Progress Report is to explore fundamental concepts of POM‐substrate composites, based on the most relevant literature examples. We will start with a comprehensive analysis of chemical anchoring modes and state‐of‐the‐art synthetic approaches (Section 2). We will then describe experimental and theoretical methods which enable us to gain insights into the intricate interplay between POM and substrate with a focus on recently established techniques as well as in situ/in operando methods relevant for rationalizing the material performance in applications including (electro/photo)catalysis and energy storage (Section 3). We will then explore the recent literature of POM immobilization on functional substrates with a focus on metals, metal oxides and semiconductors (Section 4). In particular, we will discuss details of POM‐substrate effects observed in the literature (a summary of relevant articles is further presented in **Table** **1**), explore their impact on the chemical and electronic properties of the resulting composite and establish their implications for reactivity, stability, and applications. Based on this analysis, we provide an outlook on emerging areas of academic and technological importance where supported POM systems could in the future lead to new applications (Section 5).

**Table 1:** Summary table for the surface‐attached POM composites showing their components (POM and substrate), synthetic protocol, type of interaction, used applications and year published. Examples are sorted primarily according to the type and nature of substrates used starting from inorganic semiconductors (metal oxides, metal sulfides, carbon nitrides) and followed by microporous materials (MOFs, ZIFs, COFs), polymers, nanocarbons (CNTs, graphene) and metals

POMs	Substrate	Preparation method	Type of interaction	Application	Year	Ref.
[H_3_PW_12_O_40_]	TiO_2_	Impregnation	N/A	Photosensing of acetone	2015	[103]
[H_3_PMo_12_O_40_]	TiO_2_	Impregnation	N/A	Hydrodeoxygenation and alkylation of phenolics	2017	[104]
[H_9_P_2_Mo_15_V_3_]·51H_2_O and [H_6_PMo_9_V_3_O_40_]·34H_2_O	TiO_2_	LBL	N/A	MO degradation	2013	[105]
[H_3_PW_12_O_40_]	TiO_2_ NT	Electrodeposition	N/A	Nitrobenzene degradation	2014	[106]
(Bu_4_N)_4_[W_10_O_32_]	TiO_2_ (mesoporous)	In situ templated sol–gel synthesis	N/A	Aerobic oxidation of alcohols	2015	[84]
Na_7_[PW_11_O_39_]	TiO_2_ (film)	Spin coating and calcination	Covalent	Dye degradation	2004	[52]
[H_3_PW_12_O_40_]	TiO_2_/FTO	Doctor blading	N/A	Photoanode in DSSC	2016	[107]
[H_3_PW_12_O_40_]·H_2_O and [H_4_SiW_12_O_40_]·H_2_O	TiO_2_/FTO	Sol–gel and screen printing	Electrostatic	Photoanode in DSSC	2016	[108]
[H_3_PW_12_O_40_] and K_6_[P_2_W_18_O_62_]	TiO_2_/FTO	Mixing and doctor blading	N/A	Photoanode	2013	[73]
K_10_[P_2_W_17_O_61_]·20H_2_O or K_6_[P_2_W_18_O_62_]·14H_2_O	TiO_2_/FTO	Electrodeposition	Hydrogen bonding and acid basic	Smart window (electrochromic material)	2013	[109]
Cs_9_[Ru^IV^_4_O_5_(OH)(H_2_O)_4_(γ‐PW_10_O_36_)_2_] and Rb_8_K_2_[{Ru^IV^_4_(OH)_2_(H_2_O)_4_}(γ‐SiW_10_O_34_)_2_]	TiO_2_/FTO	Impregnation onto a silylated electrode	Electrostatic	Photoanode for water splitting	2015	[110]
K_3_[Ag(H_2_O)(H_3_PW_11_O_39_)]	TiO_2_/ITO	Electrode immersion into POM solution	N/A	Photoanode for water oxidation	2018	[111]
K*_n_*[M_4_(XW_9_O_34_)_2_] (M = Mn^2+^, Cu^2+^, Zn^2+^, X = Ge^4+^, P^5+^, As^5+^)	Ln‐doped TiO_2_ (Ln = Nd^3+^, Sm^3+^, Tb^3+^ and Dy^3+^)	Impregnation	Electrostatic and hydrogen bond	Oxidation of sulphides and alcohols	2017	[27]
(NH_4_)_42_[Mo^VI^_72_Mo^V^_60_O_372_(CH_3_COO)_30_(H_2_O)_72_]	TiO_2_, SnO_2_, WO_3_, ZnO	Mechanical mixing	N/A	Photoanode in DSSC	2016	[42]
[{Ru_4_O_4_(OH)_2_(H_2_O)_4_}(γ‐SiW_10_O_36_)_2_]^10−^	TiO_2_, SnO_2_, ZrO_2_	Impregnation	Electrostatic	Photoanode in DSSC	2013	[112]
[Co_4_(H_2_O)_2_(PW_9_O_34_)_2_]^10−^	TiO_2_/FTO, Fe_2_O_3_/FTO, BiVO_4_/FTO	LBL	Electrostatic	Photoanode for water splitting	2017	[90]
[H_3_PW_12_O_40_]	SiO_2_ (octyl and 3‐aminopropyl grafted SBA‐15)	Impregnation	Electrostatic	Ester hydrolysis reaction	2007	[51]
[H_4_SiW_12_O_40_] and K_4_[SiW_11_O_39_(O(SiOH)_2_)]	SiO_2_ (SBA‐15)	Impregnation	Covalent	—	2008	[33]
Na_7_[PW_11_O_39_], K_6_Na_2_[SiW_11_O_39_], K_6_Na_2_[GeW_11_O_39_] and K_6_Na[HBW_11_O_39_]	SiO_2_ (ordered macroporous)	In situ templated sol–gel synthesis	N/A	Photocatalytic degradation of malic acid	2002	[113]
SiW_11_	SiO_2_ (MCM‐41)	Impregnation	Electrostatic	Acid‐catalyzed synthesis of biodiesel	2013	[35]
Na_7_[H_2_LaW_10_O_36_]·32H_2_O	SiO_2_ (mesoporous modified with ionic‐liquid)	Impregnation (Ion‐exchange)	Electrostatic	Extractive catalytic oxidative desulfurization	2014	[36]
(NH_4_)_6_[P_2_Mo_18_O_62_] and (NH_4_)_6_[P_2_W_18_O_62_]_,_ [H_6_P_2_Mo_18_O_62_] and [H_6_P_2_W_18_O_62_]	SiO_2_, Al, ITO	Drop‐casting or spin‐coating	N/A	—	2018	[77]
[H_3_PMo_12_O_40_]·5H_2_O	ZrO_2_ (ordered mesoporous)	In situ templated sol–gel synthesis	Electrostatic	Oxidation of alkenes mediated by H_2_O_2_	2011	[28]
[H_3_PW_12_O_40_]·16H_2_O and [H_4_SiW_12_O_40_]·15H_2_O	ZrO_2_ (ordered mesoporous)	In situ templated sol–gel synthesis	N/A	Oxidation of alkenes mediated by H_2_O_2_	2014	[56]
Na_12_[α‐P_2_W_15_O_56_]·24H_2_O	γ‐Al_2_O_3_	Impregnation	Covalent	Oxygenation of thioethers to sulfoxides	2016	[30]
[H_3_PW_12_O_40_]	Ta_2_O_5_	In situ templated hydrothermally assisted sol–gel synthesis	Covalent	Esterification of lauric acid and the transesterification of tripalmitin	2008	[34]
[{Ru^IV^_4_(OH)_2_(H_2_O)_4_}(γ‐SiW_10_O_34_)_2_]^10‑^	Fe_2_O_3_/FTO	Impregnation onto a silylated electrode	N/A	Photoanode for water splitting	2017	[94]
[H_3_PW_12_O_40_] and K_6_[CoW_12_O_40_]	BiVO_4_/FTO	Impregnation	N/A	Photoanode for water splitting	2017	[41]
[H_3_PW_12_O_40_]	BiVO_4_/FTO	Impregnation	N/A	Photoanode for water splitting	2018	[72]
[H_3_PMo_12_O_40_]	SnO_2_/FTO	Impregnation	N/A	Photoanode	2014	[114]
(C_4_H_10_ON)_23_[HN(CH_2_CH_2_OH)_3_]_10_[H_2_Fe^III^(CN)_6_(α_2_‐P_2_W_17_O_61_Co^II^)_4_]·27H_2_O	SnO_2_ (nanorods)/ITO	Impregnation	N/A	Photoelectrochemical gas sensing of formaldehyde and methylbenzene detection	2017	[115]
[H_3_PMo_12_O_40_]	ITO	LBL	Electrostatic	Sensing	2017	[62]
K_28_Li_5_[H_7_P_8_W_48_O_184_] ·92H_2_O	ITO (amine‐functionalized)	LBL method	Electrostatic	Smart window (electrochromic device)	2018	[116]
[H_5_PMo_10_V_2_O_40_]	ZnO (within a MOF)	Impregnation	N/A	Photoelectrochemical gas sensing devices for formaldehyde	2018	[96]
[Ru_4_O_4_(OH)_2_(H_2_O)_4_(γ‐SiW_10_O_36_)_2_]^10−^	WO_3_ (with conducting polymer)	Electrodeposition	N/A	Photoanode for water splitting	2018	[117]
[(CH_3_)_4_N]_5_[PW_10_Mo_2_O_40_]^11−^·4H_2_O	Cu_2_O/FTO	Impregnation	Electrostatic	Photocathode	2013	[95]
K_7_[HNb_6_O_19_]	CdS	Hydrothermal (biomolecule mediated)	Electrostatic	Photocathode for H_2_ evolution and RhB degradation	2017	[44]
[SiW_12_O_40_]^4−^, [PW_12_O_40_]^3−^, [PMo_12_O_40_]^3−^	CdS (mesoporous)	In situ templated synthesis	N/A	Aerobic oxidation of benzyl alcohols	2014	[118]
K_7_[HNb_6_O_19_]	Cd_0.65_Zn_0.35_S	Impregnation	N/A	Photocathode for H_2_ evolution	2014	[40]
[SiW_11_O_39_]^8−^	g‐C_3_N_4_ (functionalized)	Impregnation	Covalent	Photocatalytic H_2_O_2_ production	2018	[48]
[SiW_12_O_40_]^4−^, [PW_12_O_40_]^3−^, [PMo_12_O_40_]^3−^	g‐C_3_N_4_ (exfoliated)	Impregnation	Electrostatic and hydrogen bonding	Photocatalytic MO degradation and water splitting	2017	[43]
[PW_12_O_40_]^3−^; [PMo_12_O_40_]^3−^	g‐C_3_N_4_	Hydrothermal	N/A	Dyes and phenolics degradation	2015	[119]
Fe^III^{PO_4_[WO(O_2_)_2_]_4_}	g‐C_3_N_4_	Impregnation	Non‐covalent	MO and RhB degradation	2016	[120]
[PW_11_O_39_]^7−^	g‐C_3_N_4_ (ordered macroporous, functionalized)	Impregnation	Covalent	Photocatalytic H_2_O_2_ production	2017	[121]
[H_3_PMo_12_O_40_]_,_ [H_3_PW_12_O_40_]_,_ (NH_4_)_3_[PMo_12_O_40_]_,_ (NH_4_)_3_[PW_12_O_40_]	g‐C_3_N_4_ (mesoporous), graphitic carbon (N‐doped) and activated carbon	Impregnation	N/A	Methanol oxidation	2018	[78]
Na_10_[Co_4_(H_2_O)_2_(PW_9_O_34_)_2_]	g‐C_3_N_4_/FTO	Hydrothermal	Hydrogen bonding	Photoelectrochemical CO_2_ reduction	2017	[39]
[H_3_PMo_12_O_40_]	C_3_N_4_ NT	Hydrothermal	N/A	Electrochemical sensing	2017	[122]
[H_4_SiW_12_O_40_]	C_3_N_4_ (KOH‐modified, functionalized)	Impregnation	Covalent	Photocatalytic N_2_ fixation	2018	[123]
[Co_4_(H_2_O)_2_(PW_9_O_34_)_2_]^10−^	C_3_N_4_ (mesoporous, protonated)/ITO	Impregnation	Coordination bond	Electrocatalytic OER	2012	[47]
[H_3_PMo_12_O_40_]	MIL‐100	Hydrothermal	N/A	Electrocatalytic HER	2018	[124]
Na_10_[Co_4_(H_2_O)_2_(PW_9_O_34_)_2_]	MIL‐101(Cr)	Impregnation	Electrostatic	Photocatalytic and electrochemical OER	2016	[125]
(TBA)_7_[H_3_Co_4_(H_2_O)_2_(PW_9_O_34_)_2_]	MIL‐101 (Cr)	Impregnation	Encapsulation	Catalytic oxidation	2013	[126]
K_11_[Eu(PW_11_O_39_)_2_], (TBA)_6_[H_5_Eu(PW_11_O_39_)_2_], K_11_[Sm(PW_11_O_39_)_2_], (TBA)_8_[H_3_Sm(PW_11_O_39_)_2_]	MIL‐101 (Cr)	Impregnation	Encapsulation	Catalytic oxidation of styrene	2013	[127]
[H_3_PW_12_O_40_]	MIL‐100 (Fe)	Hydrothermal	Encapsulation	Catalytic esterification of cinnamic acid	2018	[128]
K_5_[CoW_12_O_40_]	MIL‐101 (Cr)	Hydrothermal	Encapsulation	Catalytic methanolysis of epoxides	2017	[129]
[H_3_PW_12_O_40_]	MIL‐101(Cr)‐diatomite	Impregnation	Encapsulation	Catalytic desulfurization	2018	[130]
[H_3_PW_12_O_40_]	MIL‐100 (Fe), UiO‐66, ZIF‐8	Impregnation/Hydrothermal	Encapsulation	Catalytic desulfurization	2017	[131]
[H_6_PMo_9_V_3_O_40_]	MOF‐199@SBA‐15	Hydrothermal	Encapsulation	Catalytic hydroxylation of benzene	2014	[132]
[H_3_PW_4_O_12_], [H_5_PMo_12_O_40_], [H_5_PVMo_10_O_40_], [H_5_PV_2_Mo_10_O_40_], [H_5_PV_3_Mo_10_O_40_]	MOF‐199	Hydrothermal	Encapsulation	Catalytic oxidation of benzylic alcohols	2014	[133]
Na_10_[Co_4_(H_2_O)_2_(PW_9_O_34_)_2_]	MOF‐545	Impregnation	Encapsulation	Photocatalytic OER	2018	[134]
[H_3_PMo_12_O_40_]	Co‐based cationic MOF	In situ hydrothermal synthesis	Electrostatic	Catalytic desulfurization	2015	[135]
[H_3_PMo_12_O_40_]	NENU‐5 (on carbon cloth)	Milling and then hot‐pressing	N/A	LIB	2018	[136]
[H_3_PW_12_O_40_]	NENU‐3a	In situ MOF synthesis	Encapsulation	Catalytic biodiesel production	2015	[137]
[H_5_PMo_10_V_2_O_40_]	NENU‐9	In situ MOF synthesis	Covalent	Catalytic oxidation of large alcohols	2018	[138]
[H_3_PMo_12_O_40_]	NENU‐5	In situ MOF synthesis	N/A	Sensing	2018	[139]
[H_3_PW_12_O_40_]	Zr‐BTC MOF	In situ solvothermal synthesis	encapsulation	Catalytic Friedel‐Crafts acylation of anisole	2018	[140]
[H_3_PW_12_O_40_]	NU‐1000	Impregnation/supercritical CO_2_ drying	Encapsulation	Catalytic oxidation of 2‐chloroethyl ethyl sulphide	2018	[46]
[Zn_2_(NH_2_‐BPY)_2_(HPYI)_2(_H_2_O)(CH_3_CN)][ZnW_12_O_40_]	PYI1, PYI2	Solvothermal	Electrostatic and covalent	Catalytic conversion of CO_2_ to carbonates	2015	[141]
[H_3_PW_12_O_40_]	ZIF‐67	Impregnation	Encapsulation	Photocatalytic OER	2016	[142]
[H_3_PMo_12_O_40_]	ZIF‐67	Impregnation	Encapsulation	Electrocatalytic OER	2018	[143]
[H_3_PMo_12_O_40_]	ZIF‐67	Impregnation	Encapsulation	Water splitting	2018	[144]
K_6_[CoW_12_O_40_]	ZIF‐8	Impregnation	Encapsulation	Electrocatalytic OER	2018	[31]
[H_4_PMo_11_VO_40_], [H_5_PMo_10_V_2_O_40_], [H_6_PMo_9_V_3_O_40_]	rho‐ZIF	Mechanochemical synthesis	Encapsulation	Catalytic oxidation of thioanisoles	2017	[145]
[H_5_PMo_10_V_2_O_40_]	ZIF‐8@ZnO	Impregnation	Encapsulation	Sensing	2018	[96]
[H_3_PMo_12_O_40_]	COF‐300	Impregnation	N/A	Catalytic epoxidation of olefins	2015	[146]
[H_2_W_6_O_19_]	Poly(vinylpyrrolidone)	Hydrothermal	N/A	Rewritable paper (photochromic)	2018	[147]
(TBA)*_n_*_+2_[V*_n_*M_6−_ *_n_*O_19_] (M = W(VI) or Mo(VI); *n* = 0, 1, 2)	PPy	Electrochemical polymerization	Electrostatic	Supercapacitors	2017	[64]
[H_3_PMo_12_O_40_]	PPy	Polymerization	Electrostatic	Supercapacitors	2018	[148]
(TBA)_4_[PMo_11_VO_40_]	CNT	Ultrasonication	Covalent and hydrogen bond	LIB	2016	[19]
[Co_4_(H_2_O)_2_(PW_9_O_34_)_2_]^10−^	N‐doped CNTs	Linker‐free method, by Impregnation	Electrostatic	Electro‐catalytic water oxidation	2017	[149]
(TBA)_4_[H_3_PMo_11_O_39_]	ox‐SWCNTs and rGO	Drop‐casting	Electrostatic and hydrogen bond	Sensing	2016	[150]
[H_6_Mn_3_V_18_O_42_(VO_4_)(H_2_O)_12_]	GO	Hydrothermal	Electrostatic	LIB	2018	[151]
[H_3_PMo_12_O_40_]	GO	Impregnation	Electrostatic	Electrocatalytic HER	2015	[152]
K_28_Li_5_[H_7_P_8_W_48_O_184_]	rGO	Electrochemical reduction	Electrostatic and hydrogen bond	Electrocatalytic HER	2016	[38]
[H_3_PMo_12_O_40_]	rGO	Hydrothermal	Electrostatic and hydrogen bond	Sensing	2017	[153]
K_6_[P_2_W_18_O_62_], K_12.5_Na_1.5_[NaP_5_W_30_O_110_], K_28_Li_5_[H_7_P_8_W_48_O_184_]	Reduced oxidized graphene	Electrochemical reduction	Electrostatic and hydrogen bond	Electrocatalytic HER	2018	[154]
(TBA)_3_(DMA)[(MnCl)V_12_O_32_Cl]	Graphene QD	Ultrasonication	Electrostatic and hydrogen bond	LIB	2017	[20]
[H_3_PW_12_O_40_]	Carbene nanocages	Hydrothermal	Covalent	LIB	2018	[155]
[H_3_(Cp*Rh)_4_PMo_8_O_32_], [H_5_Na_2_(Cp*Ir)_4_PMo_8_O_34_]	Ni foam	Hydrothermal	N/A	Electrocatalytic HER	2017	[93]
Na_3_[PW_12_O_40_]	Ni foam	Hydrothermal	N/A	Electrocatalytic OER	2017	[156]

BTC, 1,3,5‐benzene tricarboxylic acid; COF, covalent‐organic framework; DMA, *N*,*N*‐dimethylacetamide; DSSC, dye‐sensitized solar cells; FTO, F‐doped SnO_2_; GO, graphene oxide; HER, hydrogen evolution reaction; ITO, In‐doped SnO_2_; LBL, layer‐by‐layer; LIB, Li‐ion batteries; MO, methyl orange; MOF, metal–organic framework; MWCNTs, multi‐walled carbon nanotubes; NT, nanotube; OER, oxygen evolution reaction; PPy, polypyrrole; PYI, pyrrolidine‐2‐yl‐imidazole; QD, quantum dot; rGO, reduced graphene oxide; RhB, Rhodamine B; SWCNT, single‐walled carbon nanotubes; TBA, tetrabutylammonium hydroxide; ZIF, zeolitic imidazolate framework; in situ synthesis means that the procedure has been accomplished in the presence of the POM.

## 2 Synthetic Approaches to POM‐on‐Substrate Immobilization

**Figure 1:**
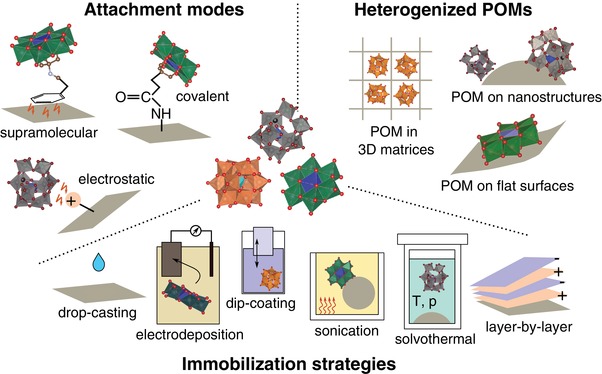
Immobilization of POM clusters. Schematic illustration of various attachment modes, substrate types, and immobilization strategies that are often used to prepare heterogenized POMs.

General POM immobilization routes have already been summarized in recent reviews.^[^ ^12^, ^17^ ^]^ Therefore, we will only briefly discuss the most commonly used synthetic concepts to deposit POMs on various heterogeneous substrates and describe the most important modes of chemical interaction between POM and support as schematically illustrated in **Figure** **1**.

### 2.1 Synthetic Approaches to POM Deposition

Various techniques have been developed to deposit POMs as individual molecules, micro‐ and nanostructured particles, layers, or bulk materials on heterogeneous substrates.^[^ ^11^, ^12^, ^18^ ^]^

#### 2.1.1 Dip‐Coating

This is the classical approach where the desired heterogeneous substrate is immersed in a solution of the POM of choice. The method is straightforward, easily scalable, and can be applied for virtually any POM‐substrate combination. However, it often suffers from reversible POM‐binding (leaching), which can lead to non‐homogeneous distribution, for example, due to POM crystallization during drying, and gives little control over the deposition process.

#### 2.1.2 Solvothermal Deposition

This route often enables mechanically and chemically stable anchoring of POM crystals on substrates at elevated temperature and pressure. These conditions facilitate POM crystallization, often in highly condensed, insoluble lattices so that leaching and reversible deposition can be overcome. However, the harsh conditions can also lead to structural re‐arrangements of sensitive POMs, and degradation into solid‐state metal oxides is possible (and can even be desirable, e.g., to fabricate highly stable materials for demanding application conditions).

#### 2.1.3 Sonication Deposition

As a more gentle alternative to solvothermal syntheses, recent studies have used sonication‐driven deposition of POMs (performed in organic solvents at room temperature/pressure).^[^ ^19^ ^]^ Variation of the sonication parameters is also established as a means of controlling particle size, shape and surface arrangement. However, recent studies also showed that structurally labile vanadates, in particular, can undergo structural rearrangements into nanostructured solid‐state oxides under sonication deposition.^[^ ^20^, ^21^, ^22^ ^]^

#### 2.1.4 Layer‐by‐Layer Assembly

This approach uses the stepwise deposition of anionic POM layers and cationic layers to build well‐defined films with tunable thickness and high stability on heterogeneous substrates. However, when thick films are targeted, this process can be time‐consuming and laborious. In addition, leaching can become an issue depending on the conditions under which the films are used.

#### 2.1.5 Drop‐Casting

This approach typically uses POM (micro/nanoparticles) dispersed in a suitable solvent which is then dropped onto the substrate. Drop‐casting is often used to modify electrode surfaces with a POM or POM composite. For electrocatalysis, often a protective Nafion layer is added on top of the POM layer to protect and stabilize the electrode.^[^ ^23^ ^]^ The method enables fast surface‐modification of electrodes; however, it gives little control over the structure and morphology of the composite layer on the electrode and can negatively affect the electrical contacting between electrode and composite.

#### 2.1.6 Electrodeposition

This method uses cyclic voltammetry of a POM‐containing homogeneous solution to deposit the POM (or POM‐composites) onto the electrode surface. It is therefore limited to conductive substrates for (photo)electrochemical applications, but benefits from controlled film growth and provides direct electrochemical information on the POM deposition process.

### 2.2 Chemical Interactions between POM and Substrate

Essentially, we can distinguish three chemical binding modes that are typically observed for POM‐substrate composites. These have been summarized earlier^[^ ^3^, ^18^, ^24^ ^]^ and are briefly described here.

#### 2.2.1 Electrostatic Anchoring

The introduction of cationic surface charges (e.g., ammonium or pyridinium groups) allows electrostatic binding of the anionic POMs. While this method is straight‐forward and can be used to anchor most POMs, it is also prone to leaching, and the stability of the anchoring depends on the strength of the electrostatic interactions, the solution pH, solvents, and the reaction conditions under which the material will be deployed.

#### 2.2.2 Covalent Anchoring

Functionalization of the substrate surface and the POM with complementary, typically organic groups is used to form covalent POM‐substrate bonds for stable and persistent anchoring. Prime examples are the formation of amide bonds using carboxylate and amine functionalization, imine bonds formed between aldehydes and amines, or 1,3‐dipolar cycloadditions (CLICK chemistry) by alkyne and azide functionalization.^[^ ^25^ ^]^ While this route gives access to stable covalent bonds, it is restricted to POMs which can be organo‐functionalized and requires multi‐step reactions and expert knowledge.

#### 2.2.3 Supramolecular Anchoring

Supramolecular interactions such as π‐stacking or hydrogen‐bonding can also be used for anchoring POMs to substrates. To this end, suitable interactions between both species need to be identified and the POM needs to be correspondingly functionalized. A prime example is the organo‐functionalization of POMs with aromatic groups (e.g., pyrene) which enable π‐stacking attachment, for example, to nanostructured carbon substrates.^[^ ^26^ ^]^

## 3 Characterization of Heterogenized POMs

Advanced experimental and theoretical methods are vital to explore the intricate interactions of POMs on substrates. In contrast to homogeneous studies, where a large range of transmission‐based spectroscopies are available, the characterization of solid, often nanostructured or amorphous samples is inherently more challenging. In addition to structural and chemical features, exploration of electronic states and their changes during operation requires a wide range of methods and their application under in situ/operando conditions. Here, we briefly describe the most important approaches and exemplify their benefits with recent literature studies.

### 3.1 Structural Characterization Techniques

The most common methods used to gain structural information of the surface‐immobilized POMs and confirm their attachment are Fourier‐transform infrared (FTIR) and UV–vis‐NIR absorption spectroscopies. Both are often performed in the solid‐state in reflectance mode using attenuated total reflectance (ATR) and diffuse‐reflectance geometries or in transmission mode using, for example, KBr‐pellets. However, it was shown by Gholamyan et al.^[^ ^27^ ^]^ that ATR, being a more surface‐sensitive method of extracting IR data, provides more information on the surface‐immobilized POM species, which is especially important if the POM loadings get small.

POMs feature two principle absorption features: In the IR, they show strong and characteristic metal–oxygen vibrational modes in the 1000–500 cm^−1^ range. In the UV‐near visible region (typically at wavelengths <450 nm), they show strong ligand‐to‐metal charge transfer (LMCT) bands; mixed‐valent POMs (e.g., featuring V^IV^ and V^V^) also show intervalence charge‐transfer bands (IVCT) in the ≈600–1200 nm region. Analyses of these characteristic signals can provide information on the structural integrity of the POM, possible POM‐substrate interactions (e.g., by shifts of the signals, or the appearance of new vibrational modes associated with substrate‐POM vibrations) as well as on the POM charge state (by changes of the IVCT bands).

**Figure 2:**
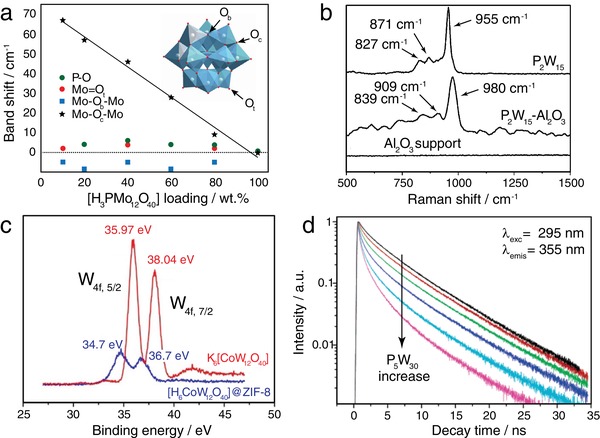
A collection of spectroscopic characterization data for POM composites. a) IR band shifts of characteristic POM bands observed by ATR‐FTIR spectroscopy for [H_3_PMo_12_O_40_] supported on rGO as a function of on the cluster loading amount. Note that 100 wt% loading corresponds to the pure POM compound. Reproduced with permission.^[^ ^29^ ^]^ Copyright 2013, American Chemical Society. b) Raman spectra of Al_2_O_3_, Na_12_[α‐P_2_W_15_O_56_]‐Al_2_O_3_ and Na_12_[α‐P_2_W_15_O_56_] (P_2_W_15_) showing the POM‐based Raman‐bands (and shifts thereof), as well as the absence of Al_2_O_3_‐based Raman bands in the corresponding region. Reproduced with permission.^[^ ^30^ ^]^ Copyright 2016, Wiley‐VCH. c) Core‐level W4f XPS spectra for K_6_[CoW_12_O_40_] and [H_6_CoW_12_O_40_]@ZIF‐8 indicating shifts in the binding energy values upon immobilization and d) fluorescence decay of human serum albumin (HSA) as a function of [NaP_5_W_30_O_110_]^14−^ (P_5_W_30_) concentration, showing the decrease in emission lifetime of HSA with increase in the amount of POM. c) Reproduced with permission.^[^ ^31^ ^]^ Copyright 2007, American Chemical Society. d) Reproduced with permission.^[^ ^32^ ^]^ Copyright 2018, Wiley‐VCH.

Only a few reports, however, provide detailed analyses of observed peak shifts. For example, Armatas et al.^[^ ^28^ ^]^ suggested that blue shifts in the UV–vis spectra of mesoporous ZrO_2_‐attached POMs can be related to quantum confinement effects, which may provide evidence of single‐molecule dispersion of the POMs. Also, Tessonnier et al. reported that the position of the Mo—O—Mo bands of [H_3_PMo_12_O_40_] is highly sensitive to the type of counter‐cation type (by examining Li^+^, K^+^, Cs^+^, etc.). Based on this, the authors suggested that the observed peak shift of this POM supported on reduced graphene oxide (rGO) is related to strong electrostatic interactions between the cluster and the protonated support that acts as the counter‐cation (**Figure** **2**a).^[^ ^29^ ^]^

Note that IR data for POM‐substrate composites are often dominated by the substrate vibrational modes, thus analysis of the POM bands can be difficult. Raman spectroscopy can provide vital complementary information regarding the attachment, associated with shifts in the stretching modes of the POM, particularly if the substrate is not Raman active in the region where POM‐based vibrational modes are observed. For example, Hong et al. detected the presence of Na_12_[α‐P_2_W_15_O_56_] on a γ‐Al_2_O_3_ support after immobilization. In addition, the authors observed strong blue shifts of the W=O stretch bands and W—O—W stretching modes in the Raman spectra of the composite (Figure 2b),^[^ ^30^ ^]^ which were associated with the strong covalent interactions between the trilacunary POM cluster and the substrate.

Magic angle spinning (MAS)‐solid‐state nuclear magnetic resonance (NMR) is an important, but less commonly used method to gain structural information on POMs on substrates. Various techniques including ^14^Si‐, ^31^P‐, and ^51^V‐NMR can be used to confirm the presence and structural integrity of POMs in substrates, and in addition can provide information on the POM‐substrate binding mode (often by observation of NMR peak shifts).^[^ ^33^, ^34^, ^35^, ^36^ ^]^ For example, Hong et al.^[^ ^30^ ^]^ proposed the covalent attachment of the trilacunary Dawson anion Na_12_[α‐P_2_W_15_O_56_] to γ‐Al_2_O_3_ by the formation of Al—O—W covalent bonds using ^31^P‐NMR. Based on the signal shifts of the two inequivalent phosphorus centers in the POM, the authors proposed a two‐step binding mechanism where electrostatic surface attachment is followed by a subsequent condensation reaction to give the covalent bonds.

X‐ray photoelectron spectroscopy (XPS) provides information on the chemical composition, oxidation states, and the local binding environment of the elements probed. It is particularly useful for POMs deposited on surfaces, as XPS is surface sensitive and examines only the top few nanometers of a given sample. Shifts in binding energies can thus be used to identify binding sites of the anchored POMs as well as the type of interaction between POM and substrate (Figure 2c). For example, Xu et al.^[^ ^34^ ^]^ investigated [H_3_PW_12_O_40_] deposited on a Ta_2_O_5_ substrate. They observed peak shifts to lower binding energies of ≈0.1–0.2 eV for the W 4f and the Ta 4f signals of the composites. This information together with IR and Raman spectroscopy allowed the authors to suggest that terminal W=O groups of [H_3_PW_12_O_40_] coordinate to the hydroxylated Ta^5+^ sites at the surface of Ta_2_O_5_ forming new Ta—O—W bonds and likely involving electron transfer from the terminal oxygen to the substrate.

As another example, Wang et al.^[^ ^37^ ^]^ and Liu et al.^[^ ^38^ ^]^ both observed that the W 4f peaks of the POM/rGO nanocomposites shifted to lower binding energies compared with those of pure [H_4_SiW_12_O_40_] and K_28_Li_5_[H_7_P_8_W_48_O_184_], respectively, indicating a strong interaction between the POMs and the rGO. The authors further assigned the spectral changes to charge transfer from POM to rGO upon immobilization, which decreases the electron density of the adjacent terminal oxygen atoms of the POMs.

Photoluminescence (PL) and time‐resolved PL (TRPL) spectroscopies are often used in materials science to evaluate charge carrier dynamics. Both can also provide insight into interfacial charge transfer processes at the POM/substrate interfaces to reveal the behavior and lifetimes of photoexcited charge carriers. In one example, Zhang et al.^[^ ^32^ ^]^ used steady‐state PL and lifetime studies to demonstrate the presence of electrostatic interactions between POMs and human serum albumin (HSA). They examined two representative POMs, the wheel‐shaped [NaP_5_W_30_O_110_]^14−^ and the α‐metatungstate anion [H_2_W_12_O_40_]^6−^, and investigated the resulting emission quenching of the HSA fluorescence dependent on the POM concentration (Figure 2d). The authors measured the lifetimes of the excited state of tryptophan within the protein, and observed a dramatic decrease in emission lifetimes, due to interactions with the POMs. They also applied Stern–Volmer analysis to the fluorescence quenching data and concluded that an electrostatic interaction is present between POMs and HSA macromolecules.

Powder X‐ray diffraction (PXRD) is crucial to analyze crystalline POM‐substrate composites.^[^ ^39^ ^]^ Typically, the absence of POM‐based XRD signals is used as an indicator of the molecular dispersion of POMs on the surface. However, further evidence is required, as the absence of such a signal can also be explained by the presence of larger, amorphous aggregates.^[^ ^40^ ^]^ In addition, PXRD can be used to detect the formation of new crystalline metal oxide phases, for example, due to degradation of POMs under operational conditions.^[^ ^41^, ^42^, ^43^, ^44^ ^]^

Difference envelope density (DED) analysis of PXRD data is a more specific technique to study host–guest relationships.^[^ ^45^ ^]^ The method uses electron density maps calculated by subtracting the envelope of the parent material from that of the composite to gain insights into structural features of the composite components. As an example, Buru et al. investigated [H_3_PW_12_O_40_]@MOF (NU‐1000) composites and used DED to identify the POM location in microporous or mesoporous channels of the substrate.^[^ ^46^ ^]^

Thermogravimetric analysis (TGA) can be used to verify the thermal stability of POM‐substrate composites, to provide an estimate of the POM loading of the composite,^[^ ^47^, ^48^ ^]^ and to reveal if the POM anchoring affects their thermal stability. In one example, Juan‐Alcaniz et al. encapsulated [H_3_PW_12_O_40_] in the micropores of the MIL‐100(Cr) MOF and used TGA to calculate the initial degree of POM incorporation that were in agreement with elemental analyses.^[^ ^49^ ^]^

Physisorption measurements can provide insights into the porosity of POM‐substrate composites and into the POM distribution. For example, Herrmann et al. characterized POM‐ionic liquids deposited on porous silica with nitrogen sorption and showed that a nanometer‐thin coating of the pores with the ionic liquid is possible without altering the large surface area of the sample.^[^ ^50^ ^]^ Only a few reports applied more sophisticated techniques based on gas sorption to analyze POM acidic sites after immobilization on a support. For example, Inumaru et al.^[^ ^51^ ^]^ used pyridine chemisorption to quantify the acidic sites of [H_3_PW_12_O_40_] anchored to mesoporous SiO_2_.

For biphasic solid–liquid applications, leaching studies are of the utmost importance, as weak POM‐substrate interactions could lead to a transfer of the POMs into the liquid reaction phase, so that separation and recycling, for example, for catalytic applications, could be problematic.^[^ ^9^ ^]^ To this end, various elemental analytic methods including inductively coupled plasma atomic emission or optical emission spectroscopy (ICP‐AES or ICP‐OES) are commonly used to quantify POM leaching into solution.^[^ ^30^, ^52^ ^]^

### 3.2 Microscopy Techniques

Microscopic techniques have become state‐of‐the‐art to gain insights into structural features of composites down to atomic resolution.

**Figure 3:**
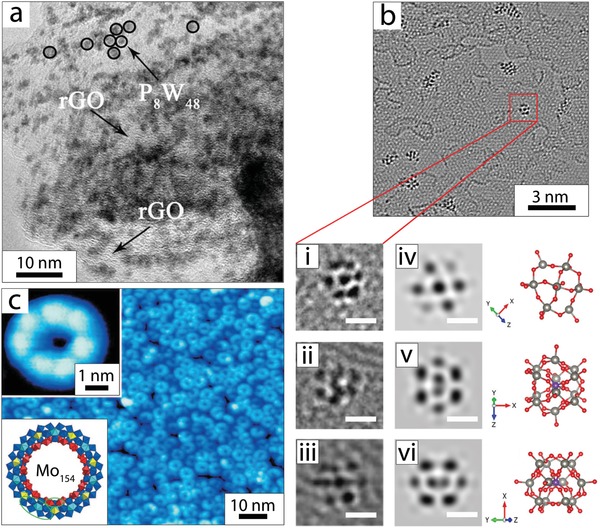
A collection of microscopy characterization data of POM composites. a) HRTEM images of the as‐prepared K_28_Li_5_[H_7_P_8_W_48_O_184_]/rGO nanocomposite, showing the individual POM anions (abbreviated here as P_8_W_48_) dispersed on the rGO surface. Reproduced with permission.^[^ ^38^ ^]^ Copyright 2016, the Royal Society of Chemistry. b) AC‐HRTEM image showing individual [H_3_PW_12_O_40_] molecules on a graphene substrate: i–iii) Magnified AC‐HRTEM images and iv–vi) image simulation of individual [H_3_PW_12_O_40_] molecules at different rotation angles along with the corresponding ball and stick structural models. Scale bars correspond to 0.5 nm. Reproduced with permission.^[^ ^55^ ^]^ Copyright 2018, the Royal Society of Chemistry. c) Constant current STM image of wheel‐shaped Mo_154_ clusters deposited on Au (111). The inset on the top: high‐resolution STM image of the Mo_154_ cluster resolving its sevenfold rotational symmetry with seven bright protrusions on the ring; inset on the bottom: ball and stick representation of Mo_154_. Reproduced with permission.^[^ ^57^ ^]^ Copyright 2011, Wiley‐VCH.

High‐resolution transmission electron microscopy (HRTEM), particularly when using aberration‐corrected (AC) low‐voltage systems,^[^ ^53^ ^]^ provides structural information down to atomic resolution with limited beam damage to the sample. HRTEM has recently been used to study metal cluster assemblies and structural rearrangements within carbon nanotubes,^[^ ^54^ ^]^ as well as to evaluate POM‐cluster distribution on solid substrates (**Figure** **3**a).^[^ ^30^, ^36^, ^38^ ^]^ A recent example is the work of Vats et al., who used AC‐HRTEM to resolve sub‐monolayer [PW_12_O_40_]^3−^ coatings on freestanding graphene, and observe surface diffusion and rotation of the POM clusters (Figure 3b).^[^ ^55^ ^]^ In contrast, scanning electron microscopy (SEM) provides structural information, typically with tens of nanometer resolution and gives insights into the composite morphology and—in combination with energy‐dispersive X‐ray spectroscopy (EDX)—can confirm the presence of POMs, observe their distribution and quantify their loadings on substrates.^[^ ^28^, ^56^ ^]^

Scanning tunneling microscopy (STM) enables structural characterization of surfaces (and molecules deposited on surfaces) at the atomic level. The instrument can be operated under vacuum as well as in air or when the sample is immersed in suitable solutions, giving access to in situ studies. STM analyses are most helpful to explore (sub)monolayer‐assemblies of POMs formed on well‐ordered substrates.^[^ ^58^ ^]^ Early examples from the 1990s have explored the supramolecular 2D ordering of POMs on model substrates, for example, highly oriented pyrolytic graphite (HOPG).^[^ ^59^ ^]^ Later, host–guest interactions between POMs and 2D frameworks deposited on gold single crystals have been explored and have allowed precise identification of the complex interactions within these systems.^[^ ^60^ ^]^ In one example, Zhong et al. demonstrated how the combination of STM and scanning tunneling spectroscopy (STS) can be used to study the electronic structure of the Na_15_[H_14_Mo^VI^_126_Mo^V^_28_O_462_(H_2_O)_70_] cluster deposited on Au (111). The authors revealed the spatial distribution of the POM HOMO and LUMO levels, and provided information on the local electronic features such as the distribution of the electronic density of states within the cluster (Figure 3c).^[^ ^57^ ^]^ Furthermore, in situ STM studies have explored the formation of POM‐based self‐assembled monolayers on gold electrodes and correlations between electrochemical studies and STM analyses have become possible.^[^ ^61^ ^]^

Atomic force microscopy (AFM) can be used to probe the surface morphology of POM composites. In contrast to electron microscopic techniques, AFM imaging is based on the intermolecular interactions of a probe with the sample surface, resulting in a 3D topography of the sample at high resolution. Using this technique, Hao et al.^[^ ^62^ ^]^ analyzed [H_3_PMo_12_O_40_]/ polyethyleneimine (PEI) composites to explore the POM distribution as well as the sample surface roughness to assess the quality of the layer‐by‐layer‐assembly.

### 3.3 Electrochemical Techniques

Electrochemical analyses are vital to understanding the performance of POMs deposited on conductive supports as used, for example, in electrocatalysis, batteries and photoelectrochemistry.

**Figure 4:**
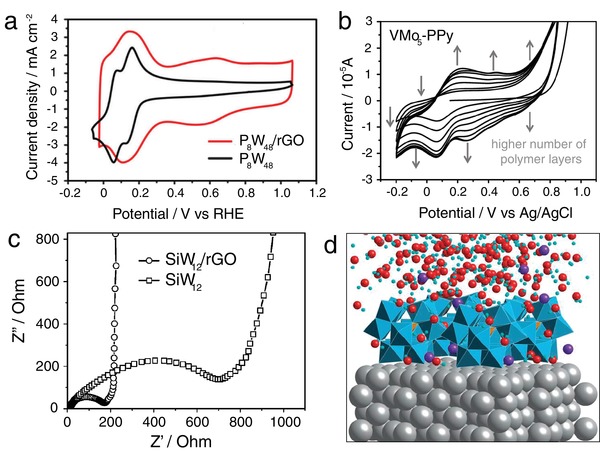
A collection of characterization data with the focus on electrochemistry and theory. a) Comparison of the cyclic voltammograms of K_28_Li_5_[H_7_P_8_W_48_O_184_]·92H_2_O (P_8_W_48_) in homogeneous solution (black) and the K_28_Li_5_[H_7_P_8_W_48_O_184_]·92H_2_O/rGO composite modified glassy carbon electrode (red) in 0.5 M H_2_SO_4_. Reproduced with permission.^[^ ^38^ ^]^ Copyright 2016, the Royal Society of Chemistry. b) Cyclic voltammograms of the electropolymerization process of pyrrole in the presence of (*n*Bu4N)_3_[VMo_5_O_19_] (VMo_5_), showing the growth of electroactive Lindqvist‐modified polypyrrole (PPy) films. Reproduced with permission.^[^ ^64^ ^]^ Copyright 2017, Wiley‐VCH. c) Comparison of the Nyquist plots of the [H_4_SiW_12_O_40_] (SiW_12_) and SiW_12_/rGO electrodes. Reproduced with permission.^[^ ^37^ ^]^ Copyright 2013, Wiley‐VCH. d) Molecular dynamics snapshot of the [SiW_12_O_40_]^4−^ anion adsorbed on Ag (100) in the presence of K^+^ ions and water molecules. Reproduced with permission.^[^ ^71^ ^]^ Copyright 2012, the Royal Society of Chemistry.

Cyclic voltammetry (CV) can be used to prove the successful anchoring of POMs on a substrate by detecting characteristic POM‐based redox waves. Changes of these signals, including shifts, absences,^[^ ^63^ ^]^ or merging of two waves can provide critical information on substrate‐POM interactions. For example, Liu et al.^[^ ^38^ ^]^ observed that the first two 4‐electron reversible reduction waves associated with the W^VI^ centers in K_28_Li_5_[H_7_P_8_W_48_O_184_] merged into a single 8‐electron wave when the POM was immobilized on rGO (**Figure** **4**a). This was attributed to the “microenvironment effect,” and was also observed in the other redox waves of the composite. One explanation for this “microenvironment effect” is that the ratio of proton to POM concentrations in the composite is different compared to that in solution. In addition, for the growth of POM‐containing films on electrodes, for example, by electropolymerization or layer‐by‐layer (LBL) techniques, CV analysis provides information on film growth and film conductivity (Figure 4b).^[^ ^63^, ^64^ ^]^ After POM immobilization on electrodes, CV can be used to monitor stability and reactivity of the POMs upon subsequent (electro)chemical reactions.^[^ ^62^, ^65^, ^66^, ^67^ ^]^ Variation of the CV scan rate^[^ ^62^, ^65^, ^68^ ^]^ or the use of a rotating disk electrode^[^ ^65^ ^]^ can allow the determination of which processes control the kinetics of an electrochemical reaction.

Electrochemically active surface area (ECSA) studies based on CV data can provide information on the electrode surface coverage with an electroactive species and enable the determination of the electrochemically active surface area. This is a facile means to compare the electrochemical activities of different composites deposited on electrodes and helps to elucidate observed reactivity differences, for example, for electrocatalytic processes.^[^ ^62^, ^69^ ^]^

Linear sweep voltammetry (LSV) in combination with rotating disc electrodes is a classical approach to studying electrocatalytic reactions.^[^ ^65^ ^]^ This analysis technique enables extraction of key electrochemical data relevant for catalysis, such as overpotentials or Tafel slopes, although we note here that bulk electrolysis is a more rigorous method to derive this data, as it provides enough time for the current to stabilize for each applied potential. LSV can, in addition, provide information on the kinetic limitations of the processes, for example, by diffusion. Many derivative electrochemical techniques based on CV/LSV are described in reference textbooks^[^ ^70^ ^]^ and are not discussed here for reasons of brevity.

Electrochemical impedance spectroscopy (EIS) provides valuable information on the charge transport properties of a composite. EIS determines the resistivity of a sample depending on an AC current frequency. A simplified electrical circuit model (the so‐called equivalent circuit) then allows extraction of different contributions to resistivity, such as charge diffusion, interfacial charge transfer, and electronic coupling resistivity. For example, EIS has been used to show that POMs deposited on reduced graphene oxide (rGO) can effectively reduce the contact and charge‐transfer resistances, leading to improved lithium‐storage performance (Figure 4c).^[^ ^37^, ^44^, ^72^, ^73^ ^]^ In addition, EIS analysis in the presence of a redox probe (e.g., ferricyanide) can provide information on the diffusion of an electroactive species and thus on the porosity of the sample, which is particularly relevant for multilayer composites.^[^ ^63^ ^]^

Electrochemical quartz crystal microbalance (EQCM) studies can be used to monitor film formation or film dissolution during electrochemical cycling. It can, for example, be used to follow the electropolymerization growth of redox‐active polymer‐POM composites. Dong et al.^[^ ^66^ ^]^ demonstrated this and explored the redox‐induced movement of ions into POM‐based multilayer films using in situ time‐resolved EQCM. The report also suggests that ion diffusion can be correlated to the redox‐processes of the POM so that ionic mobility and POM redox‐activity can be directly linked.

### 3.4 Computational Techniques

Density functional theory (DFT) calculations are invaluable to assess structural and electronic features of POMs including frontier orbital energies, redox‐processes as well as reaction mechanisms. Their critical input in POM chemistry has recently been reviewed.^[^ ^74^ ^]^ To explore larger systems, such as POM molecules deposited on surfaces, periodic DFT (pDFT), as well as molecular dynamics (MD) calculations, can be of value. These techniques have been used to rationalize the behavior of Keggin‐POMs deposited on Au(100) model surfaces.^[^ ^74^ ^]^ The combination of both calculation approaches enabled rationalization of the electronic materials properties (by pDFT) and in addition, solvent and counterion effects were explored using MD simulations (Figure 4d).^[^ ^71^ ^]^ Note that these complex calculations require well‐defined (i.e., model) surfaces and that computational effort scales with the number of atoms in the system, thus effectively coupling the system size to the development of computing power.^[^ ^74^ ^]^

## 4 Substrate Effects and Functional Properties

The choice of substrate for POM deposition is primarily defined by the target application. In the case of acid‐, base‐ or redox‐catalysis, especially in liquid phase, inorganic substrates that feature a large, accessible surface area—such as nanostructured carbons and porous metal oxides—have been used to support POM species. More recently, advanced materials including MOFs, ZIFs, COFs, and zeolites have attracted enormous attention as active supports for POM immobilization, for they combine a well‐defined porous environment with the potential of having added catalytic functionalities. On the other hand, in applications where conductive supports are desired—such as in electrocatalysis or optoelectronics—conductive polymers, metals or nanocarbons that offer high electrical conductivity are the materials of choice. In contrast, for application where light‐harvesting and charge‐separation are the key factors—including photo(electro)catalysis and photovoltaics—the main focus has been on semiconducting materials that feature tunable band gap, electronic structure and (photo)conductivity.

Depending on the type and chemistry of the substrate, different synthetic protocols and attachment strategies need to be used to deposit a given POM on a chosen support, as has been summarized in Section 2. Carbon‐based and organic/polymeric materials typically offer a wide range of chemical tunability to allow for controllable attachment of POM species and desired type of interaction (e.g., covalent or supramolecular). In contrast, the deposition of POM anions on inorganic substrates may benefit from the more hydrophilic nature of the latter. For example, metal oxides have very similar chemistry and thus can be combined with POM clusters via direct covalent bonding by the formation of M—O—M bonds, without the need for organic linking groups.^[^ ^75^ ^]^

However, the immobilization of POMs on heterogeneous substrates is not only driven by the basic physicochemical properties of the supports, but also by their added functionality or synergistic functions. For example, in the field of heterogeneous thermal catalysis, it has long been established that the type of catalytic support has a strong effect on the overall performance: this is often referred to as “metal‐support interactions,” or “substrate effects.”^[^ ^76^ ^]^ In the simplest case, such substrate effects originate from both electronic and structural changes caused by strong catalyst‐support interactions. Prime examples are polarization effects, where the heterogeneous substrate leads to charge density changes within the catalyst, and the decoration of the metal nanoparticles through mobile molecular substrate species. Both cases strongly affect the reactivity and selectivity of the catalysts.

So far only a few works have investigated the extent and role of synergistic effects in POM‐based composites. Some recent studies, however, highlight that the changes in POM structure and reactivity are strongly dependent on the type of substrate and mode of interaction between both components. For example, Argitis et al.^[^ ^77^ ^]^ recently explored how the redox properties of immobilized Wells–Dawson ammonium salts (NH_4_)_6_[P_2_Mo_18_O_62_], (NH_4_)_6_[P_2_W_18_O_62_] and their respective Keggin heteropolyacids [H_3_PMo_12_O_40_] and [H_3_PW_12_O_40_] are affected by the type of substrates used. The authors compared metallic Al, dielectric SiO_2_ and semiconducting ITO substrates and followed (by UV–vis and XPS spectroscopy) the degree of reduction of the immobilized POMs. Spontaneous redox reactions between POM and both Al and ITO substrates were observed under ambient conditions, with the extent of the POM reduction being dependent on the relative position of its LUMO with respect to the substrate Fermi level and also on the presence of ammonium counter ions. On the other hand, no spontaneous charge transfer was observed for the dielectric SiO_2_ substrate, highlighting that POM deposition on substrates can be used to modify the POM redox state upon immobilization.

In the following sections, we will discuss—based on the most relevant literature examples summarized in Table 1—how immobilization can affect the structure and reactivity of heterogenized POMs with respect to: i) nature and extent of POM‐substrate interactions; ii) impact of the attachment on electronic properties; and iii) its implication on the stability of the POM species.

### 4.1 Active Sites and Synergies

Specific physical and chemical interactions between POM and substrate often result in new, sometimes unexpected reactivity. This section will explore how the interplay between both components affects physical and electronic structures of POMs on heterogeneous supports, resulting in new catalytic functions.

#### 4.1.1 Tuning Bronsted Acidity

**Figure 5:**
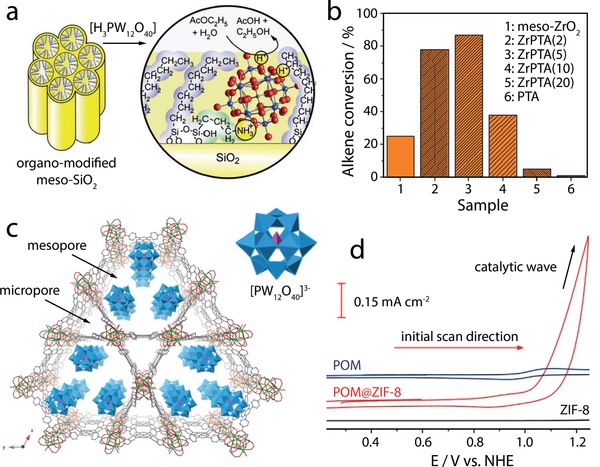
Active sites and synergy. a) Schematic illustration of the structure of the POM‐SiO_2_ catalysts: octyl and 3‐aminopropyl groups were subsequently grafted on the pore walls of mesoporous silica, followed by immobilization of [H_3_PW_12_O_40_] molecules inside the pores. Reproduced with permission.^[^ ^51^ ^]^ Copyright 2007, Wiley‐VCH. b) Oxidation of 1,1‐diphenyl‐2‐ methylpropene catalyzed by heterogeneous mesoporous ZrO_2_ (meso‐ZrO_2_), homogeneous [H_3_PW_12_O_40_] (PTA) and the ZrPTA (wt%) composites with POM loadings of 2, 5, 10, and 20 wt%. Reproduced with permission.^[^ ^56^ ^]^ Copyright 2014, the Royal Society of Chemistry. c) Structural representation of one possible POM location in [H_3_PW_12_O_40_]@NU‐1000 composite inferred from DED analysis. Light blue octahedra, WO_6_; pink tetrahedra, PO_4_. Reproduced with permission.^[^ ^46^ ^]^ Copyright 2018, the Royal Society of Chemistry. d) Cyclic voltammograms of ZIF‐8, POM@ZIF‐8 modified glassy carbon electrode and POM (K_6_[CoW_12_O_40_]) dissolved in aqueous solution at pH of 1.9 in the potential range of water oxidation. Reproduced with permission.^[^ ^31^ ^]^ Copyright 2018, Wiley‐VCH.

An early example of a substrate effect was reported by Inumaru et al,^[^ ^51^ ^]^ who deposited [H_3_PW_12_O_40_] on organo‐modified mesoporous SiO_2_. The substrate had been pre‐functionalized with 3‐aminopropyl and *n*‐octyl groups to explore the binding behavior of the POM (**Figure** **5**a). The authors explored the catalytic activity of the POM‐based composite in the acid‐catalyzed hydrolysis of ethyl acetate. As a reference reaction, they studied the same catalytic process using [H_3_PW_12_O_40_] under homogeneous conditions. The study gave a 3.5‐fold increase in catalytic activity (per acidic proton) of the composite compared with the homogeneous reference. The authors assigned this to the embedding of the acidic protons of POMs within a hydrophobic environment formed by the aliphatic chains. The grafting has thus enabled facile access of the reactant molecules to the acidic sites. However, questions remain as to why the heterogeneous system outperforms the homogeneous reference where lower diffusion limitation and faster kinetics are expected. One explanation for the excellent catalytic performance, in this case, involves a potential influence of the substrate on the structural and electronic properties of the POM, for example, via charge transfer or distortion of M—O polyhedra, which the authors, however, did not discuss.

More recent studies have used heterogeneous substrates to modulate the acidity of immobilized heteropolyacids: Barteau et al.^[^ ^78^ ^]^ studied how the nitrogen content in various supports affects the Bronsted acidity of [H_3_PMo_12_O_40_] as well as its catalytic activity for methanol oxidation. The group investigated activated carbon, N‐doped graphitic carbon with low (2%) and high (19 %) nitrogen content as well as mesoporous graphitic carbon nitride with an N content of 53%. For the supports containing NH_2_ groups, the authors observed the formation of the corresponding POM ammonium salts (NH_4_)_3_[PMo_12_O_40_] based on PXRD analyses and assigned this to the extraction of the amine groups from the N‐doped substrates. Butene chemisorption experiments were used to evaluate the number of acidic POM sites: for activated carbon and 2% N‐doped graphitic carbon, no change in the number of acidic sites was observed, while for the supports with higher N‐content, a significant reduction in the number of acidic sites was found. This change was also reflected in MeOH oxidation—a catalytic test reaction that supposedly involves interactions between the MeOH and protons from the catalyst.^[^ ^79^ ^]^ Here, the increasing N content of the support led to a dramatic decrease of overall catalytic reactivity, although a higher selectivity for products following the oxidative pathway versus those obtained through the dehydration pathway was observed for the substrates with the highest N loading. These results show that the substrate composition can modulate the Bronsted acidity of the immobilized POM, thus promoting either oxidation or acid‐catalyzed reactions when both pathways are in competition for a given reactant.

#### 4.1.2 Selectivity

Armatas et al.^[^ ^28^ ^]^ investigated the role of the substrate in tuning the selectivity of heterogenized POM catalysts. The authors used an in situ immobilization protocol, where mesoporous ZrO_2_ was synthesized (by a sol–gel route) in the presence of [H_3_PMo_12_O_40_]. The samples showed a homogeneous distribution and no notable aggregation of the POM species after deposition. Subsequently, the authors compared the catalytic oxidations of alkenes to alcohols and ketones by the composites with the homogeneous POM. Notably, both reactions showed similar turnover frequencies (TOFs, calculated based on the moles of POM present). This either suggests that the immobilized POMs were fully accessible for the substrate and showed similar reactivity to the homogeneous system. Alternatively, if only a part of the POMs were accessible for the substrate, this would mean that they showed higher activity compared with the homogeneous system. The authors also demonstrated that the heterogeneous reaction produced significantly fewer side products compared with the homogeneous reaction, highlighting that immobilization can be used to optimize reaction selectivities.

Similar selectivity differences were also observed by Hong et al.^[^ ^30^ ^]^ who studied the selective oxygenation of thioethers using H_2_O_2_ as oxidant and Na_12_[α‐P_2_W_15_O_56_] deposited electrostatically on Al_2_O_3_ as heterogeneous catalyst. The authors reported that in the presence of the composite catalyst, conversion and selectivity were improved considerably compared with the homogeneous system. A similar application (i.e., oxidation of methyl phenyl sulfide to methyl phenyl sulfoxide or to methyl phenyl sulfone) has been targeted by Gholamyan et al.,^[^ ^27^ ^]^ who immobilized [Mn_4_(XW_9_O_34_)_2_]^10−^ (X = P^5+^, As^5+^) on TiO_2_ nanoparticles. Compared to TiO_2_ or POM alone, only the POM/TiO_2_ composite was able to catalytically convert methyl phenyl sulfide to methyl phenyl sulfoxide by a surprisingly high chemo‐selectivity and at high rates. The authors concluded that heterogenization was necessary to tune the active sites; however, more work is needed to understand and explain this performance.

#### 4.1.3 Triple‐Phase Boundaries

Another key aspect of immobilized POM reactivity changes is the formation of so‐called triple‐phase boundaries, where three components (i.e., POM, substrate and reagent solution) meet and enable new adsorption and dissociation sites and thus novel synergistic reactivity. Skliri et al.^[^ ^56^ ^]^ explored this concept in POM catalysis and reported a significantly improved catalytic alkene oxidation by [H_3_PW_12_O_40_] and [H_4_SiW_12_O_40_] when deposited on a mesoporous ZrO_2_ (Figure 5b). The authors suggested that the POMs and surface ZrO*_x_*(OH)*_y_* species act as cooperative reaction sites that enable adsorption and activation of the C=C bonds of the 1,1‐diphenyl‐2‐methylpropene. In addition, based on gas chromatography and NMR analyses, the authors noted different reaction selectivities for the ZrO_2_‐supported POMs compared to the pure substrate or POM, which provides further evidence of a possible change in the nature or activity of the reactive sites upon composite formation.

Based on these examples, it can be further envisaged that a controlled deposition of POMs on substrates can lead both to selective blocking of specific reaction sites as well as the creation of new reaction sites, thus providing a tool to tune selectivities toward a particular catalytic path.

#### 4.1.4 Confinement Effects

The use of well‐defined porous environments can be a facile means to control POM incorporation and thereby enable or disable specific catalytic reaction pathways, for example, using size selectivity of transition state geometry stabilization.^[^ ^80^, ^81^ ^]^

Buru et al.^[^ ^46^ ^]^ have examined how the location of POM clusters within a MOF affects their reactivity. The authors examined the composite [H_3_PW_12_O_40_]@NU‐1000 and developed a route to selectively deposit the [H_3_PW_12_O_40_] units inside the mesopores or micropores of the MOF. As a model reaction, they explored the selective partial oxidation of 2‐chloroethyl ethyl sulfide. In all cases, the composites were more active toward sulfide oxidation compared to the POM or MOF alone. Notably, higher reaction rates and higher product selectivity were observed when the [H_3_PW_12_O_40_] species were immobilized inside the larger mesopores (Figure 5c), where reactant diffusion and pore access are less hindered. The data illustrate that the ability to site‐ and size‐selectively deposit POMs within complex porous materials enables precise control over their catalytic activity, so that the POM environment and the POM‐MOF‐substrate interactions play key roles in tuning the overall reactivity of the clusters.^[^ ^38^, ^82^ ^]^

Mukhopadhyay et al.^[^ ^31^ ^]^ have recently transferred this concept to the oxygen evolution reaction. To this end, the authors encapsulated the Keggin cluster K_6_[CoW_12_O_40_] as catalytic sites into the cavities of ZIF‐8 (ZIF = zeolitic imidazolate framework). Electrochemical studies of the composite showed significantly enhanced OER catalytic activity compared with the pure POM in both acidic and neutral electrolyte (Figure 5d). The authors suggested that this enhancement is due to a “microenvironment effect” within the ZIF cavities, where an alternative, hydrogen‐bond‐assisted water oxidation mechanism becomes possible. However, thus far, the exact role of the POM anion in the water oxidation process needs further investigation to understand its true role in the catalytic process, particularly since Co‐containing POMs are known to decompose into solid‐state cobalt oxo hydroxides under some water oxidation conditions.^[^ ^83^ ^]^

#### 4.1.5 New Synergistic Reactivity

Following the previous example, it has been also reported, that heterogenization of POM species not only defines a particular reaction path, but can also enable a new one. In a recent example, Symeonidis et al. evaluated the catalytic aerobic alcohol photooxidation by homogeneous [(Bu_4_N)_4_W_10_O_32_] and compared this with the same POM immobilized on TiO_2_.^[^ ^84^ ^]^ Their analyses of product distribution and reaction kinetics indicated that POM anchoring has enabled an alternative reaction pathway where TiO_2_ is involved in a charge transfer to facilitate the oxidation step. The authors showed that the homogeneous system is dominated by a hydrogen atom transfer (HAT) mechanism while the heterogenized system proceeds predominantly via an electron transfer (ET) mechanism, leading to the observed differences in reactivity.

### 4.2 Electronic Interactions with the Substrate

Another important aspect of POM immobilization is the extent of the electronic interaction between the substrate and the POM clusters. This section will elucidate how interfacial charge transfer can be used and tuned to develop demanding applications in photo(electro)catalysis and solar cells.

#### 4.2.1 POM as an Electron Reservoir

The capability of polyoxometalates to act as reservoirs that can reversibly store electrons while maintaining their structure has been used for a number of applications where efficient electron–hole separation and transfer are of advantage.^[^ ^85^, ^86^ ^]^

**Figure 6:**
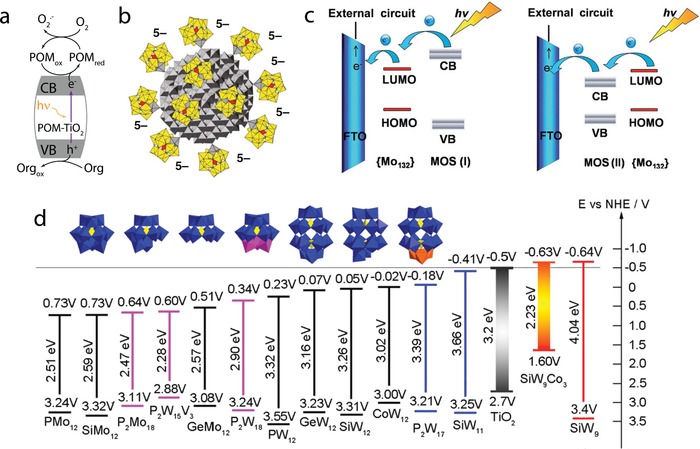
The choice of substrate and POM. a) Scheme explaining the synergistic effect between POMs and TiO_2_ leading to efficient photocatalytic organic dye degradation. When the composite is irradiated with UV light, the photoexcited electrons transfer from the conduction band (CB) of TiO_2_ to the POM, which acts as an electron acceptor and inhibits electron–hole recombination. This enables the hole to react with H_2_O‐generating OH^•^ radicals, which in turn photo‐oxidize the dye (here represented as “Org”). The POM returns to its initial state by transferring the electrons to O_2_ molecules present in the buffer. Reproduced with permission.^[^ ^87^ ^]^ Copyright 2003, the Royal Society of Chemistry. b) Scale model showing the relative sizes and distribution of [α‐PW_11_O_39_Fe] ligands decorating the surface of α‐Fe_2_O_3_ core. Reproduced with permission.^[^ ^88^ ^]^ Copyright 2018, Nature Springer. c) Scheme showing how the cluster (NH_4_)_42_[Mo^VI^_72_Mo^V^_60_O_372_(CH_3_COO)_30_(H_2_O)_72_] (abbreviated here as {Mo_132_}) can act either as an electron acceptor or an electron donor depending on the relative position of its LUMO and the conduction band of the metal oxide semiconductor (MOS). On the left, the conduction band of the semiconductor is higher than the LUMO of the POM and the electron transfer happens from the semiconductor to the POM. The opposite situation is depicted on the right. Reproduced with permission.^[^ ^42^ ^]^ Copyright 2016, the Royal Society of Chemistry. d) Diagram showing the band gaps and LUMO and HOMO redox potentials versus normal hydrogen electrode (NHE) calculated from cyclic voltammetry and diffuse reflectance spectroscopy for 14 POMs to compare with the energy levels of TiO_2_. This comparison allowed the authors to choose K_6_H_4_[α‐SiW_9_O_37_Co_3_(H_2_O)_3_]·17H_2_O (here represented as SiW_9_Co_3_) as the most suitable POM to combine with TiO_2_ for DSSC photoanode preparation owing to its small bandgap and low‐lying LUMO. Reproduced with permission.^[^ ^89^ ^]^ Copyright 2015, American Chemical Society.

The first heterogeneous photoactive POM‐TiO_2_ hybrid was reported in 2003.^[^ ^87^ ^]^ In this pioneering work the authors prepared Na_7_[PW_11_O_39_‐TiO_2_], K_6_[Na_2_SiW_11_O_39_]‐TiO_2_ and K_6_Na_2_[GeW_11_O_39_]‐TiO_2_ composites by an in situ immobilization route using polystyrene spheres as templates. Detailed analyses provided evidence of the structural integrity of the POM after immobilization, and based on IR‐data, the authors suggested a covalent anchoring mode by the formation of Ti—O—W bonds. The composites were tested for organic dye photodegradation under UV irradiation and gave significantly higher photodegradation rates compared with the pure TiO_2_ or pure POM. This was explained by a synergistic action that gave rise to a composite material in which photoelectrons are transferred from the TiO_2_ conduction band to the POM LUMO, thus preventing e^−^/h^+^ recombination (**Figure** **6**a). According to the authors, the highly oxidative hole can then migrate to the TiO_2_ surface to react with H_2_O and generate OH^•^ radicals. The authors also proposed that the significant reactivity improvement is based on the covalent bonding between both catalysts, as this could not be achieved by simple physical mixing of the components. However, the work did not provide experimental evidence on the state and distribution of the POM species on the oxide surface.

In one of the most recent examples, Chakraborty et al.^[^ ^88^ ^]^ demonstrated that the oxidatively inert heteropolytungstate Na_7_[α‐PW_11_O_39_]·12H_2_O can be used for covalent surface modification of small hematite (α‐Fe_2_O_3_) nanocrystals (Figure 6b), forming purely inorganic core–shell nanoparticles that are soluble in water over a wide pH range (from 2.5 to 8). The authors further demonstrated the ability of the hematite core to act as a visible light absorber, while the POM clusters can promote charge separation, subsequently leading to sustained water oxidation at the iron centers. The authors showed that the POM effectively prevented degradation of the iron oxide surface and also inhibited Fe_2_O_3_ particle agglomeration/precipitation during the catalytic process.

In general, most semiconductors suffer from fast electron–hole recombination, leading to lower catalytic turnover rates or poorer optoelectronic performance. Modification of a semiconductor surface with species that effectively extract/trap electrons or holes has, therefore, become an established procedure to optimize charge separation and the resulting performance. In the following section, we will discuss the key requirements that define the extent and direction of the charge transfer in semiconductors‐POM composites.

#### 4.2.2 Charge Transfer

Whether the POM acts as an electron donor or electron acceptor largely depends on the d‐electron configuration and relative position of its frontier orbitals with respect to the valence and conduction bands of the semiconductor. The principle was nicely illustrated by Kang and co‐workers who studied mixed‐valence Keplerate‐type POMs (NH_4_)_42_[Mo^VI^_72_Mo^V^_60_O_372_(CH_3_COO)_30_(H_2_O)_72_] containing Mo(V) and Mo(VI) centers which were deposited on various semiconducting supports.^[^ ^42^ ^]^ Based on the analysis of the HOMO‐LUMO levels of the cluster (using CV, UV–vis and UPS spectroscopy), the authors concluded that the POM can potentially act as either the electron donor or acceptor, depending on the oxide band structure (Figure 6c). As a proof of concept, they performed photocurrent measurements, EIS and PL quenching studies, and revealed that the POM acts as an electron acceptor in TiO_2_ and ZnO composites, and as an electron donor in SnO_2_ and WO_3_ composites as would be expected for the respective position of the energy bands.

The electronic structure of semiconductor‐POM composite components not only defines the direction of the charge transfer, but also has a pronounced impact on the resulting performance. This principle was recently illustrated by Jeon et al,^[^ ^90^ ^]^ who used a layer by layer (LbL) method to prepare POM‐semiconductor assemblies as photoanodes for photoelectrochemical water oxidation. In this study, two virtually isostructural POM water oxidation catalysts, Na_10_[Co_4_(H_2_O)_2_(VW_9_O_34_)_2_] and Na_10_[Co_4_(H_2_O)_2_(PW_9_O_34_)_2_] were tested, and under homogeneous electrochemical conditions, significantly higher turnover numbers (TONs) have been observed for the V‐containing POM.^[^ ^91^ ^][^ ^92^ ^]^ In contrast, when embedded in a hematite photoanode, the P‐containing POM showed higher water oxidation performance under photoelectrochemical conditions based on photocurrents. This intriguing reactivity is thus far unexplained and requires further study.

This example demonstrates that great care has to be taken when choosing suitable POMs and support materials for a specific application, while the understanding of the electronic junction between the components is key to rationally design the POM‐based composites.

#### 4.2.3 Electronic Matching and Band Alignment

When targeting POM‐semiconductor composites capable of charge‐transfer, for example, for photoelectrochemistry of redox catalysis, one needs to carefully align the POM and semiconductor energy levels in order to maximize charge extraction and charge utilization.

In one example, Wang et al.^[^ ^89^ ^]^ recently illustrated that the performance of a POM‐semiconductor composite can be anticipated based on the band‐orbital alignment of the components. The authors used CV in combination with diffuse‐reflectance UV–vis spectroscopy to study the electronic structure of 13 POMs (Figure 6d). Based on this data, they determined which POM would be most suitable in combination with TiO_2_ to improve the photoanode performance in dye‐sensitized solar cells (DSSCs). Based on the small HOMO‐LUMO gap and low‐lying LUMO, the authors chose the well‐matched K_6_H_4_[α‐SiW_9_O_37_Co_3_(H_2_O)_3_]·17H_2_O to prepare a POM‐TiO_2_ hybrid film grown by LBL. The composite showed a significant increase in power conversion efficiency compared with POM‐free reference samples, thus highlighting that a knowledge‐based materials design approach in POM‐semiconductor development is possible.

**Figure 7:**
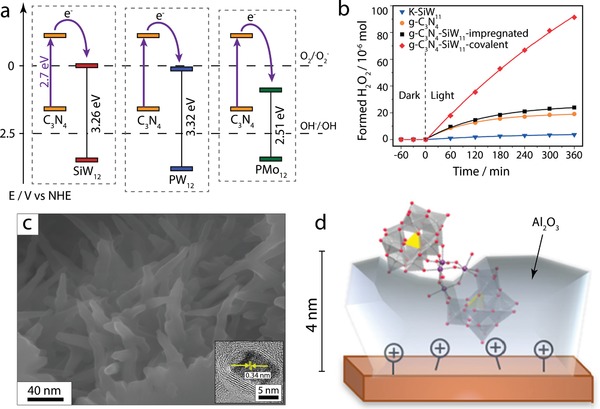
The choice of substrate and POM and composite stability. a) Scheme showing the energy levels of [H_4_SiW_12_O_40_], [H_3_PW_12_O_40_] and [H_3_PMo_12_O_40_] (abbreviated here as SiW_12_, PW_12_, and PMo_12_) with respect to that of g‐C_3_N_4_ and the redox values of the O_2_/^•^O_2_^−^ and OH^−^/OH to explain why the combination of [H_3_PMo_12_O_40_] with g‐C_3_N_4_ does not result in any synergistic effect. Reproduced with permission.^[^ ^43^ ^]^ Copyright 2017, the Royal Society of Chemistry. b) Photocatalytic data in methanol/water mixtures over one hour corresponding to H_2_O_2_ formation over K_8_[SiW_11_O_39_] (K‐SiW_11_), g‐C_3_N_4_, electrostatically bound K_8_[SiW_11_O_39_]/g‐C_3_N_4_ and covalently bound K_8_[SiW_11_O_39_]/g‐C_3_N_4_. Reproduced with permission.^[^ ^48^ ^]^ Copyright 2018, the Royal Society of Chemistry. c) SEM image of hydrothermally obtained needle‐like POM‐derived nanostructures on Ni foam surface, and HRTEM in the inset that established the presence of MoO_2_ nanoparticles trapped in a polymeric carbon over the Ni foam. Reproduced with permission.^[^ ^93^ ^]^ Copyright 2017, American Chemical Society. d) Illustration showing how the right thickness of the Al_2_O_3_ protective layer could prevent POM detachment from the substrate without blocking its active sites. Reproduced with permission.^[^ ^94^ ^]^ Copyright 2017, American Chemical Society.

The concept of energy level matching is also important for POM‐semiconductor composites for light‐driven redox‐catalysis. An instructive example of this concept was reported by Yan et al.^[^ ^43^ ^]^ The group immobilized three model POMs [H_4_SiW_12_O_40_], [H_3_PW_12_O_40_], and [H_3_PMo_12_O_40_] on graphitic carbon nitride (g‐C_3_N_4_) and explored the photooxidative methyl orange degradation by superoxide (^•^O_2_^−^) radical anions formed by one‐electron reduction of O_2_. The observed activities decreased in the order [H_4_SiW_12_O_40_]@g‐C_3_N_4_ > [H_3_PW_12_O_40_]@g‐C_3_N_4_ > [H_3_PMo_12_O_40_]@g‐C_3_N_4_ ≈ g‐C_3_N_4_. This trend can be directly correlated to the LUMO energies of the POMs. As shown in **Figure** **7**a, the LUMO energies of the three POMs are lower than the conduction band of g‐C_3_N_4_, thus enabling the transfer of a photoexcited electron from the semiconductor to the POM and consequently preventing electron–hole recombination. As the LUMOs of [H_4_SiW_12_O_40_] and [H_3_PW_12_O_40_] are close to the redox potential of the O_2_/^•^O_2_^−^ redox couple, they can easily transfer the electron to the oxygen, resulting in increased superoxide formation (and higher dye degradation). On the other hand, the LUMO of [H_3_PMo_12_O_40_] is significantly more positive than the O_2_/^•^O_2_^−^ redox potential, so that this POM essentially acts as a deep electron trap, which prevents the electron transfer to the oxygen. This negative effect counteracts any catalytic improvement based on the reduced electron–hole recombination, and the overall effect is that the photocatalytic performance of the composite is similar to that of the substrate on its own.

#### 4.2.4 Nature of Interface

As discussed in Section 2, the binding mode of immobilized POMs has a significant impact on the resulting composite properties. Covalent attachment is often considered superior as it can give rise to stronger substrate effects based on the electronic coupling between the components. This, in turn, can be used to tune and optimize composite performance.

The effect of different POM binding modes to the substrate was illustrated in a landmark study by Zhao et al.^[^ ^48^ ^]^ The group investigated g‐C_3_N_4_ functionalized with the lacunary Keggin POM K_8_[SiW_11_O_39_] for light‐driven H_2_O_2_ production (by reduction of O_2_). The authors compared covalent versus electrostatic immobilization routes and showed that the covalent bonding can result in significantly increased H_2_O_2_ production rates (+105% compared with the non‐modified g‐C_3_N_4_) and high selectivity (>80% toward H_2_O_2_) as shown in Figure 7b. In contrast, the electrostatically immobilized composites showed only a small increase in H_2_O_2_ formation rate (+10%) and low selectivity (<25% toward H_2_O_2_). Furthermore, the authors reported that the light‐induced decomposition of H_2_O_2_ was higher for the electrostatic composite compared with the native g‐C_3_N_4_ and the covalent composite. This intriguing example highlights the importance of covalent linkage between POM and SC to modulate and optimize chemical reactivity and could be a blueprint for many other applications in (photo)electrocatalysis, energy conversion and small molecule activation.

### 4.3 Stability of the POM Attachment

One of the primary reasons for POM heterogenization is to prevent POM aggregation and degradation and to enhance their long‐term stability. Despite recent advances, POM leaching and the potential transformation into solid‐state metal oxides under operational conditions still remain unsolved issues. This is particularly challenging for applications where the composite is in contact with solvents and electrolytes or exposed to light and/or electric bias. The main reasons for the limited stability of POM‐substrate composites can be summarized as i) the (photo)corrosion of the substrate surface, ii) POM degradation by undesired structural changes; and iii) POM detachment from the surface.

One example that illustrates the first point is the work by Zhang et al.^[^ ^95^ ^]^ The authors used Cu_2_O, a semiconductor that exhibits a promising bandgap for sunlight absorption but tends to corrode in aqueous media. When the authors deposited (NMe_4_)_5_[PW_10_Mo_2_O_40_]·4H_2_O on Cu_2_O, the initial improvement observed in the photocurrent was lost after a few minutes, highlighting how principally well‐working systems can quickly degrade if suboptimal POM‐support combinations are chosen.

Generally, the fate of both, POM and substrate, under operation is critical to understanding the long‐term performance of a given system. Presently, however, post‐operational studies are often out‐of‐scope for publications, as most reports typically focus on materials design and application studies. A noteworthy exception is the work by Singh et al.,^[^ ^93^ ^]^ who have reported significant chemical changes of the deposited POMs under electrocatalytic hydrogen evolution reaction conditions. The authors used needlelike Ru‐ and Ir‐functionalized octamolybdate clusters ([H_3_(Cp*Rh)_4_PMo_8_O_32_] and [H_5_Na_2_(Cp*Ir)_4_PMo_8_O_34_] where Cp* stands for pentamethylcyclopentadienyl) deposited on Ni foam electrodes as model catalysts. During electrochemical HER in alkaline solution, both composites were chemically reduced to give M/MoO_2_ (M = Rh and Ir) nanocomposites (Figure 7c), which showed significantly higher electrocatalytic activity than the POM precursors. This example illustrates that structural evolution of POMs and substrate under harsh application conditions, that is, light illumination, electrical bias or harsh solvents, requires careful attention. It also shows that structural and chemical changes in POMs can be used as a tool to develop more active, stable catalysts that overcome intrinsic challenges of molecular POMs. As such, POMs are vital as single‐source precursors where the stoichiometry of the individual elements can be tuned together with their deposition by mild solution processing routes.

Several strategies can be used to tackle the ubiquitous challenge of POM leaching. One approach is based on the “trapping” of POM clusters within porous structures such as MOFs, ZIFs or COFs which combine high surface areas and well‐defined micropore structures with chemical tunability. The possibility of covalent modifications can further be of benefit to stabilize POM clusters in their molecular form. Conceptually, such composite systems can be further combined with other components without losing their synergistic properties. This has recently been demonstrated by Wang et al. who deposited thin layers of ZIF‐incorporated [H_5_PMo_10_V_2_O_40_] clusters onto ZnO and used this three‐component composite for selective and sensitive detection of small gas molecules.^[^ ^96^ ^]^

Another viable strategy is to use protective coatings in order to prevent POM species from leaching as well as from transformation under reaction conditions. This approach has recently been demonstrated by Lauinger et al.^[^ ^94^ ^]^ who succeeded at stabilizing a Ru‐based POM catalyst deposited on Fe_2_O_3_ photoanodes by adding an Al_2_O_3_ protective layer. The authors showed that modulation of the Al_2_O_3_ layer thickness is key for optimum performance: while very thin films did not result in significant catalyst stabilization, too thick films resulted in the catalyst blockage, resulting in poor performance, here illustrated for the photoelectrochemical water oxidation (Figure 7d).

## 5 Outlook

This Progress Report illustrates that the deposition of POMs on heterogeneous substrates opens an enormous range of application‐driven materials design opportunities, where the unique properties of POMs and functional substrates can be synergistically combined, leading to new reaction paths and unprecedented, sometimes unexpected reactivities. Research has focused initially on heterogeneous thermal catalysis, while more recently, the attention has shifted toward applications in energy conversion/storage with a focus on electrochemical and photo(electro)chemical applications. However, as exemplified in Section 4, irrespective of the final application, the type of substrate and POM need to be carefully chosen in order to enable and maximize synergistic functions and be able to tune POM‐substrate interactions. For that, new concepts in POM chemistry, as well as new possibilities in substrate design, need to be merged into novel composite design strategies for emerging applications. Some of these applications are described below based on recent pioneering studies.

Vasilopoulou et al.^[^ ^100^ ^]^ showed the potential of POMs for use in optoelectronic devices, such as OLEDs and OPVs, where the introduction of a POM electron injection layer (EIL) as cathode interlayer has led to significant device performance enhancements. The authors attributed this to the enhanced electron injection/extraction efficiency and reduced recombination losses, highlighting that further tuning of the POM electronic structure could be a facile means of optimizing performance.

**Figure 8:**
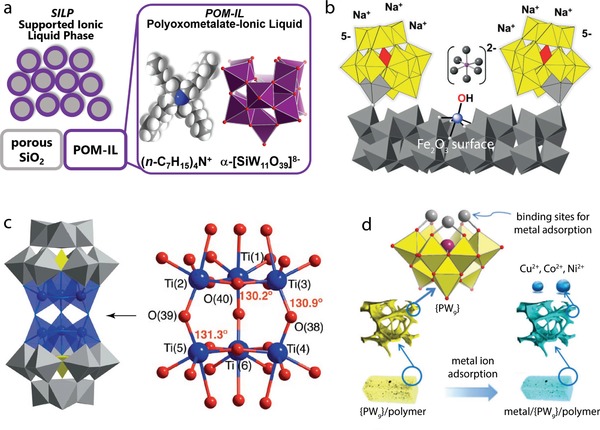
Outlook. a) Polyoxometalate‐supported ionic liquid phases (POM‐SILPs) based on water‐immiscible POM‐ILs supported on porous silica used for water purification. Reproduced with permission.^[^ ^50^ ^]^ Copyright 2017, Wiley‐VCH. b) Polyhedral presentation of the α‐Fe_2_O_3_ surface of [α‐PW_11_O_39_Fe^III^]^4−^‐O‐ligated hematite core complex with one Fe‐OH reactive site highlighted in blue (Fe^III^) and red (‐OH). Two bound POM ligands and one [H_3_I^VII^O_6_]^2−^ anion are drawn to scale. Reproduced with permission.^[^ ^97^ ^]^ Copyright 2019, Wiley‐VCH. c) Structural representation of the tri‐titanium(IV)‐substituted dimer, [(α‐1,2,3‐ PW_9_Ti_3_O_37_)_2_O_3_]^12−^. Reproduced with permission.^[^ ^98^ ^]^ Copyright 2013, Wiley‐VCH. d) Schematic illustration of the POM‐modified 3D‐printed ABS (acrylonitrile butadiene styrene) copolymer substrates used for heavy metal removal by the cation binding sites of the lacunary [α‐PW_9_O_34_]^9−^ (abbreviated here as {PW_9_}). Reproduced with permission.^[^ ^99^ ^]^ Copyright 2018, the Royal Society of Chemistry.

In the field of water‐purification, Herrmann et al.^[^ ^50^ ^]^ recently developed multi‐pollutant filtration composites by depositing water‐insoluble polyoxometalate‐ionic liquids (POM‐ILs) on highly porous silica. The composite SILPs (supported ionic liquid phases) were capable of removing organic, inorganic and biological pollutants from water by simple filtration. It is generally possible to tune their binding properties by modifying the cation and anion components of the ionic liquid, which promised interesting future developments in this field (**Figure** **8**a).

POMs can be used to create hybrid systems that bridge the field of homogeneous and heterogeneous catalysis. This concept has been explored by Chakraborty et al.^[^ ^97^ ^]^ who investigated Na_7_[α‐PW_11_O_39_]·12H_2_O, K_9_[α‐AlW_11_O_39_] and K_10_[α_2_‐P_2_W_17_O_61_], decorated Fe_2_O_3_ nanocrystals and were able to elucidate the mechanism of visible‐light‐driven water oxidation of solid‐state Fe_2_O_3_ highlighting strong differences in rate‐limiting steps of the photocatalytic process in comparison to the operation of hematite‐based electrocatalysts (Figure 8b).

Another emerging research area is the development of molecular analogs of classical semiconductor supports which can be combined with POMs on a molecular level. This exciting field has recently been exemplified for titanium oxide clusters as a model system for TiO_2_ surfaces: the combination of Ti‐O molecular clusters with polyoxometalates has already been conceptually demonstrated, while the ramifications of these findings and new applications are waiting to be explored (Figure 8c).^[^ ^98^, ^101^ ^]^

A grand challenge is the merging of substrate design by 3D printing with the capability to embed POMs into the resulting devices. Pioneering studies have already shown the post‐functionalization of 3D printed organic polymers,^[^ ^102^ ^]^ and POMs have been deposited on 3D‐printed porous structures, leading to functional devices for water purification (Figure 8d).^[^ ^99^ ^]^ Given the rapid developments in 3D printing, future substrate design can look beyond 3D printed organic polymers, and POM‐composites based on 3D‐printed ceramics, metal oxides and metals can be envisaged.

In addition to developing new POM‐based functional composites and expanding their field of applications, the community needs to overcome the lack of fundamental understanding of the POM‐substrate interactions and the resulting synergy. One of the most promising future developments will involve the rationalization of the often complex performance and stability of POM‐substrate composites during the material performance. The use of techniques that provide quantitative insights into the composite structure and performance under operation is critical for the future development of the field. This is particularly important for demanding applications, such as photo‐, electro‐, and thermal catalysis, battery applications, and photovoltaics, where high energy input and harsh chemical conditions can considerably affect the components and may lead to degradation. In particular, the use of coupled techniques, for example, spectro‐electrochemical methods, time‐resolved spectroscopic methods as well as in situ/operando techniques and theoretical studies will enable understanding of the desired and undesired processes occurring within the composites under application conditions.

Further, it is critical to develop and standardize strategies of POM immobilization based on various modes of interaction allowing for reversible or irreversible POM binding. One important development will be to compare electrostatic and covalent bonding types for the same POM‐support composites with regard to the observed reactivity differences. Such studies are urgently needed to gain a better understanding of the underlying POM‐support interaction including geometric, structural and electronic changes upon binding, as well as the extent of the electronic communication between the components, which are important for a number of applications.

In summary, POM‐substrate composites have and will enable new applications at the forefront of chemical research and materials design, and while the combinations between POMs and substrates are almost unlimited, a clear understanding of the performance and limitations of each component together with the ability to link these components in a controlled fashion forms the basis to further develop this field. The aim in the future shall be to develop a knowledge‐based materials design concept to replace purely empirical screening methods.

## Conflict of Interest

The authors declare no conflict of interest.
